# Geospatial Analysis on the Distributions of Tobacco Smoking and Alcohol Drinking in India

**DOI:** 10.1371/journal.pone.0102416

**Published:** 2014-07-15

**Authors:** Sze Hang Fu, Prabhat Jha, Prakash C. Gupta, Rajesh Kumar, Rajesh Dikshit, Dhirendra Sinha

**Affiliations:** 1 Centre for Global Health Research, Li Ka Shing Knowledge Institute, St. Michael’s Hospital, University of Toronto, Toronto, Ontario, Canada; 2 Dalla Lana School of Public Health, University of Toronto, Toronto, Ontario, Canada; 3 Healis-Sekhsaria Institute of Public Health, Navi Mumbai, India; 4 School of Public Health, Post Graduate Institute of Medical Education and Research, Chandigarh, India; 5 Department of Epidemiology, Tata Memorial Hospital, Mumbai, India; 6 South East Asia Regional Office, World Health Organization, New Delhi, India; The National Institute for Health Innovation, New Zealand

## Abstract

**Background:**

Tobacco smoking and binge alcohol drinking are two of the leading risk factors for premature mortality worldwide. In India, studies have examined the geographic distributions of tobacco smoking and alcohol drinking only at the state-level; sub-state variations and the spatial association between the two consumptions are poorly understood.

**Methodology:**

We used data from the Special Fertility and Mortality Survey conducted in 1998 to examine the geographic distributions of tobacco smoking and alcohol drinking at the district and postal code levels. We used kriging interpolation to generate smoking and drinking distributions at the postal code level. We also examined spatial autocorrelations and identified spatial clusters of high and low prevalence of smoking and drinking. Finally, we used bivariate analyses to examine the spatial correlations between smoking and drinking, and between cigarette and bidi smoking.

**Results:**

There was a high prevalence of any smoking in the central and northeastern states, and a high prevalence of any drinking in Himachal Pradesh, Arunachal Pradesh, and eastern Madhya Pradesh. Spatial clusters of early smoking (started smoking before age 20) were identified in the central states. Cigarette and bidi smoking showed distinctly different geographic patterns, with high levels of cigarette smoking in the northeastern states and high levels of bidi smoking in the central states. The geographic pattern of bidi smoking was similar to early smoking. Cigarette smoking was spatially associated with any drinking. Smoking prevalences in 1998 were correlated with prevalences in 2004 at the district level and 2010 at the state level.

**Conclusion:**

These results along with earlier evidence on the complementarities between tobacco smoking and alcohol drinking suggest that local public health action on smoking might also help to reduce alcohol consumption, and vice versa. Surveys that properly represent tobacco and alcohol consumptions at the district level are recommended.

## Introduction

The World Health Organization has identified tobacco smoking and binge alcohol drinking as two of the leading risk factors for premature mortality worldwide [1], [2]. In India, 14% per cent of the population above age 15 smoke tobacco (24% for men and 3% for women) [3]. Smoking is already responsible for about 1 in 5 deaths for men and 1 in 20 deaths for women at ages 30–69 [4]. India has among the lowest alcohol use per capita in the world, but reports growth in alcohol sales [5], [6]. More importantly, male alcohol drinking in India is characterized by problematic drinking (e.g. binge-drinking), which has negative health and social impacts on the consumers and their families [5], [7].

Most studies examined tobacco smoking and alcohol drinking behaviours separately among the Indian population. Few studies which examined the concurrent use of tobacco and alcohol found that smokers have a higher likelihood to drink alcohol than non-smokers [8], and vice versa [9], [10].

Understanding the geographic distributions of tobacco smoking and alcohol drinking, along with the determinants contributing to their use, is essential for developing targeted tobacco and alcohol control policies. Previous studies have examined the distributions of tobacco smoking [11]–[13] and alcohol drinking [14] in India at the state level separately. State level analysis can potentially mask the sub-state spatial heterogeneity, especially in the bigger states (e.g. 17 Indian states have populations greater than 25 million [15]). Implementation of tobacco and alcohol control policies is the responsibility of the state government [6], [16], thus studying the distributions of tobacco smoking and alcohol drinking as well as their spatial association at the sub-state level is desirable.

Advances in geographic information system (GIS) technology enable analysis of public health phenomena by taking “space” into account [17]. GIS is an integrated set of tools that allow both the analytical manipulation and the visual presentation of spatial data [17], [18]. Epidemiologic studies have employed GIS methods for disease mapping and cluster detection [19]–[21], and although these types of research often focus on diseases, GIS methods can also study risk factors for diseases.

Here, we utilized GIS methods to analyze the distributions of tobacco smoking and alcohol drinking in India. Our objective was to identify sub-state regions with significantly high and low prevalence of smoking and drinking. We also explored whether smoking and drinking were spatially correlated.

## Methods

### Dataset

The Special Fertility and Mortality Survey (SFMS) was a one-time study conducted in February 1998 [22]. The SFMS covered over one million nationally-representative households, based on the Registrar General of India’s Sample Registration System (SRS) [22], [23]. The SRS aims to provide reliable estimates of fertility and mortality at the state and national levels for rural and urban areas separately [24]. The SFMS interviewed household heads to provide various health indicators for the household residents, including their tobacco smoking and alcohol drinking behaviours. They were asked “does ‘household member’ smoke/drink alcohol”, “age at which ‘household member’ started smoking/drinking”, “what does he/she smoke: cigarette, bidi”, and “how frequently ‘household member’ drinks alcohol: daily, 3–4 times a week, once a fortnight, once a month, less frequently”. These definitions are similar to the National Family Health Survey (NFHS) 1998–1999 (“Does ‘household member’ smoke tobacco/drink alcohol?”) [25] and the Global Adult Tobacco Survey (GATS) 2009–2010 (“Do you currently smoke tobacco on a daily basis, less than daily, or not at all?”) [3]. The GATS survey interviewed respondents directly while the earlier surveys relied on proxy responses, usually from the head of the household.

We focused on males between 30 and 69 years old, as this age group comprises the majority of smokers (80%) and drinkers (83%), similar to the results from other surveys [11], [26], [27]. Among Indian females, only about 3% smoke regularly [3], [11] and 1% drink regularly [27], [28], thus females were excluded from the analysis. We analyzed the following smoking and drinking variables: any smoking, early smoking, cigarette smoking, bidi smoking, and any alcohol drinking. Excluding the missing records or those reporting initiation of smoking before age 10 years, the mean age of initiating any smoking, cigarette smoking, and bidi smoking was 21 (SD 4.8), 23 (SD 4.7), and 20 (SD 4.6), respectively. Therefore, we defined early smoking as those who initiated smoking before age 20. The mean age of initiating any alcohol drinking was 24 (SD 5.4).

The SFMS covered 445 out of the 467 districts from the 1991 Census. Three island districts were excluded. The geographic locations of the SRS units were further geocoded to the associated Indian postal codes, allowing analyses at the district and postal code levels.

### Geospatial analyses

Three GIS methods were used to analyse the geographic patterns of tobacco smoking and alcohol drinking and identify areas with high and low prevalence. 1) ordinary kriging interpolation was used to describe the prevalence at the postal code level, 2) Moran’s I was used to measure the overall spatial autocorrelation at the district level, 3) and the local indicators of spatial association (LISA) statistic was used to identify spatial clusters at the district level. These methods are described next, and detailed explanations can be found in Appendix S1.

Kriging is a technique that generates an estimated interpolation surface from a set of data points by incorporating the inference from the spatial structure of data points [29], [30]. First, the study population at the SRS units was aggregated to the Indian postal code locations. Crude prevalences (i.e. without age standardization) were calculated for the smoking and drinking variables at the postal code locations. The kriging procedure used the calculated crude prevalence to create an interpolation surface for the entire country. Kriging was implemented using ArcGIS software (ESRI, 2011. Environmental Systems Research Institute, http://www.esri.com/software/arcgis/). Results from kriging interpolation allowed us to validate the calculated prevalence at the district level using the SFMS data, which was designed for state level analysis [24].

Moran’s I is an index that measures the “global” spatial autocorrelation of values over the entire dataset [29]. An index score derived from the calculation indicates the strength of spatial autocorrelation of the values, with a score of 0.3 or more and −0.3 or less indicating relatively strong positive and strong negative autocorrelations, respectively [29], [31]. While Moran’s I determines the overall spatial autocorrelation of a variable, it does not provide a measure for spatial clusters. Hence, we used the LISA statistics to identify spatial clusters of the smoking and drinking variables. The LISA statistic gives an indication of the extent of significant spatial clustering of similar or dissimilar values around a spatial feature [32]. Four types of spatial associations can be derived from this statistic, with high-high (HH) and low-low (LL) types for spatial clustering of similar values, and high-low (HL) and low-high (LH) types for spatial clustering of dissimilar values, that is, spatial outliers [31]. For example, the high-high type indicates that a district with high prevalence is surrounded by neighbouring districts also with high prevalence; while low-high type indicates that a district with low prevalence is surrounded by neighbouring districts with high prevalence. We used the first order queen spatial weight for identifying neighbours for the spatial features. The first order queen weight defines neighbours as districts that share either a common border or a vertex with a given district. Preliminary analysis showed that the first order queen weight produced superior results to higher order weights (data not shown).

Two types of Moran’s I and LISA analyses were performed: univariate and bivariate. Using the district-level age standardized prevalence (Appendix S1 **)**, we performed univariate Moran’s I and LISA to examine the spatial autocorrelation and spatial clusters, respectively, for each smoking and drinking variable. We used bivariate Moran’s I and LISA to examine the spatial associations between smoking and drinking, as well as between cigarette and bid smoking.

A permutation approach was used to assess the statistical significance of the Moran’s I and LISA results (p<0.05). The permutation approach produced a reference distribution for significance testing by randomly shuffling the actual data values over space a given number of times [33]. Moran’s I and LISA statistics were calculated using GeoDa software [34]. Results from both kriging interpolation and LISA analysis were visualized using ArcGIS software.

## Results

Among men aged 30–69 years in 1998, 40.9% were smokers and 18.7% were drinkers; among smokers, 40.6% were early smokers (before age 20) (Table 1). For smoking types, six states were excluded due to greater than 20% missing data on smoking types–Haryana, Bihar, Sikkim, Meghalaya, Mizoram, and Andhra Pradesh. In the remaining states, 39.7% of men aged 30–69 years were smokers, among which 7.9% were cigarette smokers and 29.3% were bidi smokers, while 2.5% of the records had missing data for smoking type. At the postal code level, about 7% of the study population was excluded as they could not be aggregated to postal code locations. Nevertheless, the prevalence for both smoking and drinking status at the two geographic levels remained similar.

**Table 1:** Prevalence of smoking and drinking among men age 30–69 years.

	District level			Postal code level		
	# of observations	Population (n)	Prevalence (%)	# of observations	Population (n)	Prevalence (%)
**Study population**	–	1,031,625	100	–	959,411	100
Any smokers	422,353	1,031,625	40.9	394,719	959,411	41.1
Cigarette smokers†	66,244	837,433†	7.9	60,908	783,426†	7.8
Bidi smokers†	245,274	837,433†	29.3	231,164	783,426†	29.5
Early smokers (before age 20)‡	170,162	419,419‡	40.6	158,835	391,980‡	40.5
Any drinkers	192,515	1,031,625	18.7	179,392	959,411	18.7

† Six states (Haryana, Bihar, Sikkim, Meghalaya, Mizoram, and Andhra Pradesh) were excluded from the analysis for smoking type as responses were not provided for majority of the smokers (>20%) from these states.

‡ 2934 and 2739 records from the smoking population at district and postal code level, respectively, were excluded due to invalid information for the resident’s age at which he started smoking.

**Figure 1:**
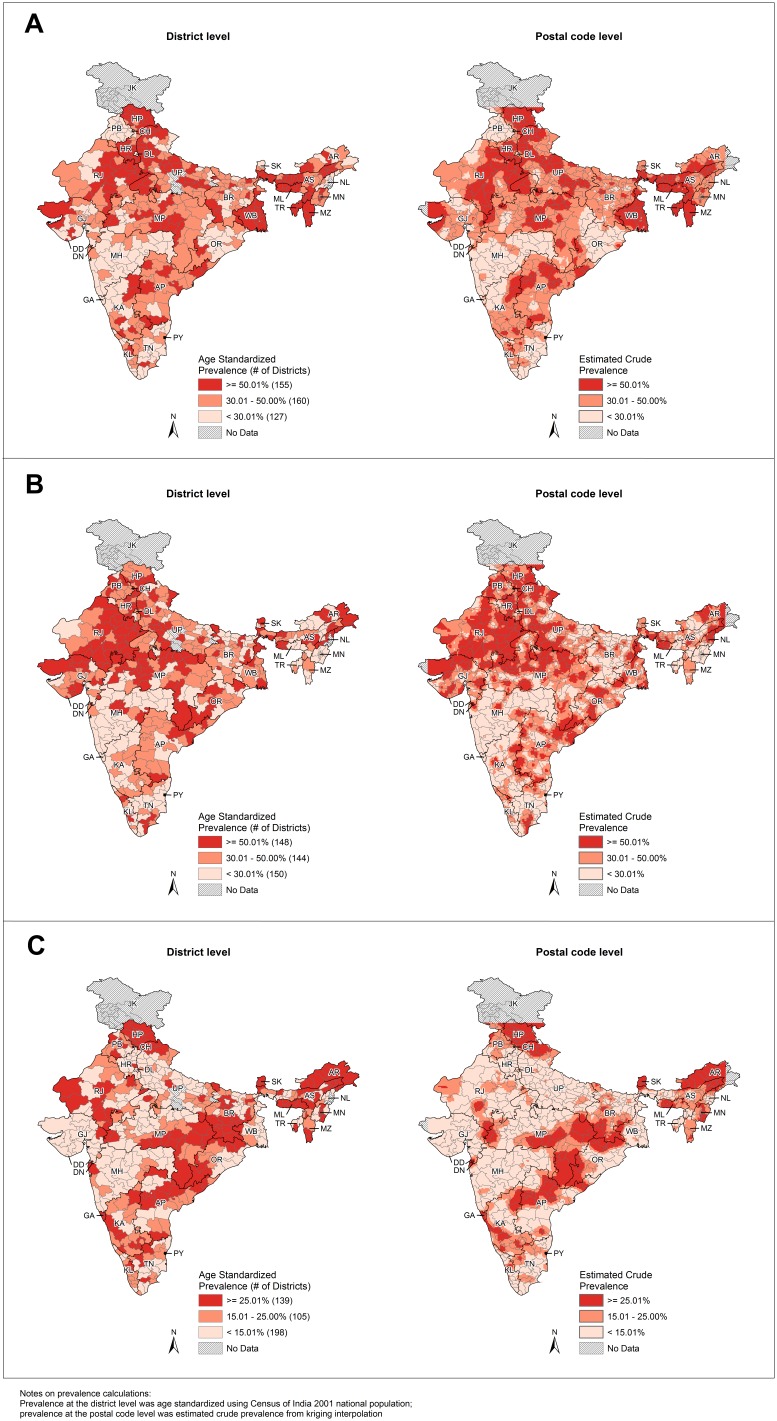
Smoking and drinking prevalence at the district and postal code levels. Prevalence at district level was age standardized using Census of India 2001 national population; prevalence at postal code level was estimated crude prevalence from kriging interpolation. State abbreviations: AP - Andhra Pradesh, AR - Arunachal Pradesh, AS - Assam, BR - Bihar, CH - Chandigarh, DD - Daman and Diu, DL - Delhi, DN - Dadra & Nagar Haveli, GA - Goa, GJ - Gujarat, HP - Himachal Pradesh, HR - Haryana, JK - Jammu & Kashmir, KA - Karnataka, KL - Kerala, MG - Meghalaya, MH - Maharashtra, MN - Manipur, MP - Madhya Pradesh, MZ - Mizoram, NL - Nagaland, OR - Orissa, PB - Punjab, PD - Pondicherry, RJ - Rajasthan, SK - Sikkim, TN - Tamil Nadu, TR - Tripura, UP - Uttar Pradesh, WB - West Bengal. **A.** Any smoking prevalence at district and postal code levels. **B.** Early smoking (before age 20) prevalence at district and postal code levels. **C.** Any drinking prevalence at district and postal code levels.

The geographic distributions for both smoking and drinking were similar between the district and postal code levels in India (Figure 1A–C, Figure S1D–E). Kriging interpolation identified the sub-district level patterns that were concealed in the district level maps. It also helped to provide estimations for districts not covered by the SFMS, such as those in Uttar Pradesh. The prevalence of smoking was high in the central, northern (except Punjab), and the northeastern states, while the prevalence was lower in Punjab, Maharashtra and Orissa (Figure 1A). Some neighbouring states showed contrasting results (e.g. high prevalence in Madhya Pradesh vs. low prevalence in Maharashtra). Prevalence of early smoking (before age 20) was high in the central and northern states (Figure 1B). Although Punjab had low smoking prevalence, it had high prevalence of early smoking (before age 20). Cigarette and bidi smoking showed distinctly different geographic patterns: high cigarette smoking prevalence was found in the southern and northeastern states (Figure S1D); while high bidi smoking prevalence was found in the central and northern (except Punjab) states (Figure S1E). West Bengal had high prevalence for both cigarette and bidi smoking. The geographic pattern of bidi smoking was similar to early smoking (before age 20). High prevalence of any drinking was located in Himachal Pradesh, parts of the northeastern states, and the central eastern part of the country (Figure 1C).

In Moran’s I analysis, all smoking and drinking variables were statistically significant (p<0.01) and had relatively strong autocorrelations (index scores close to or more than 0.3) (Table 2). Bivariate Moran’s I results showed that cigarette smoking was positively correlated to any drinking (index score = 0.119, p<0.01) and negatively correlated to bidi smoking (index score = −0.112, p<0.05) (Table 3). However, any smoking did not show any significant spatial correlation with any drinking (index score = 0.031, p = 0.14) or drinking more than 3 days a week (data not shown).

**Table 2:** Univariate global Moran’s I for smoking and drinking variables.

Variable	Moran’s I
Any smoking	0.4204**
Cigarette smoking	0.5849**
Bidi smoking	0.4620**
Early smoking (before age 20)	0.2798**
Any drinking	0.3707**

**p<0.01.

**Table 3:** Bivariate global Moran’s I for smoking versus drinking, and cigarette smoking versus bidi smoking.

Variable	Moran’s I
Any smoking *vs.* any drinking	0.0308
Cigarette smoking *vs.* any drinking	0.1190**
Bidi smoking *vs.* any drinking	−0.0525
Cigarette smoking *vs.* Bidi smoking	−0.1115*

*p<0.05;

**p<0.01.

**Figure 2:**
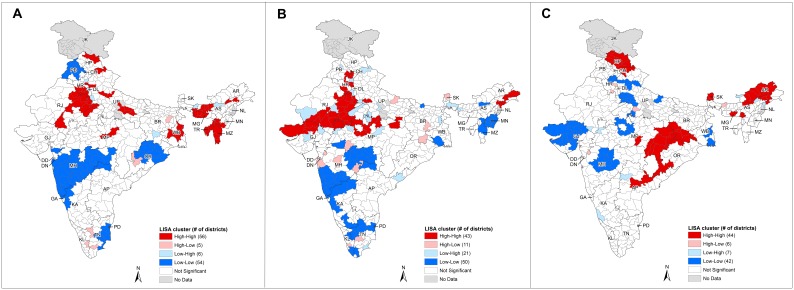
LISA cluster maps for smoking and drinking prevalence at the district level. State abbreviations: AP - Andhra Pradesh, AR - Arunachal Pradesh, AS - Assam, BR - Bihar, CH - Chandigarh, DD - Daman and Diu, DL - Delhi, DN - Dadra & Nagar Haveli, GA - Goa, GJ - Gujarat, HP - Himachal Pradesh, HR - Haryana, JK - Jammu & Kashmir, KA - Karnataka, KL - Kerala, MG - Meghalaya, MH - Maharashtra, MN - Manipur, MP - Madhya Pradesh, MZ - Mizoram, NL - Nagaland, OR - Orissa, PB - Punjab, PD - Pondicherry, RJ - Rajasthan, SK - Sikkim, TN - Tamil Nadu, TR - Tripura, UP - Uttar Pradesh, WB - West Bengal. **A.** LISA cluster map for any smoking prevalence. **B.** LISA cluster map for early smoking (before age 20) prevalence. **C.** LISA cluster map for any drinking prevalence.

The univariate LISA results highlighted districts with significantly high prevalence as high-high clusters and low prevalence as low-low clusters (Figure 2A–C, Figure S2D–E). Spatial outliers, specifically high-low and low-high types, also existed in the maps. Compared to the high-high and low-low clusters, fewer districts belonged to the high-low and low-high types, and these outliers did not often neighbour other outliers in clusters. For early smoking (before age 20) and cigarette smoking, outliers were found near the boundary shared by Rajasthan, Uttar Pradesh, and Madhya Pradesh, and they were surrounded by spatial clusters in the adjacent regions (Figure 2B, Figure S2D).

**Figure 3:**
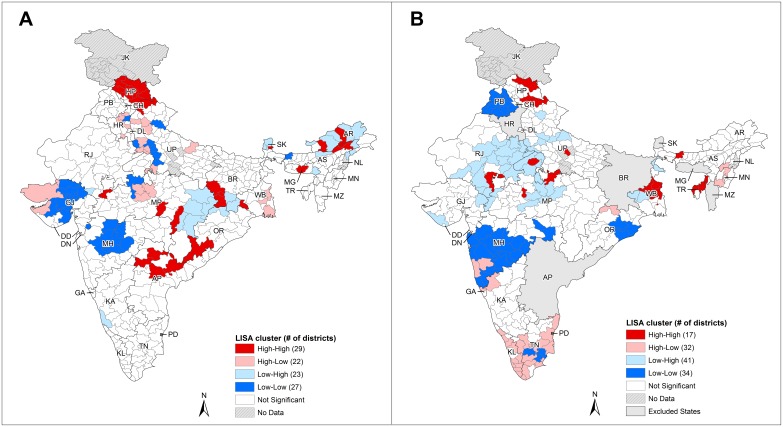
Bivariate LISA cluster maps. State abbreviations: AP - Andhra Pradesh, AR - Arunachal Pradesh, AS - Assam, BR - Bihar, CH - Chandigarh, DD - Daman and Diu, DL - Delhi, DN - Dadra & Nagar Haveli, GA - Goa, GJ - Gujarat, HP - Himachal Pradesh, HR - Haryana, JK - Jammu & Kashmir, KA - Karnataka, KL - Kerala, MG - Meghalaya, MH - Maharashtra, MN - Manipur, MP - Madhya Pradesh, MZ - Mizoram, NL - Nagaland, OR - Orissa, PB - Punjab, PD - Pondicherry, RJ - Rajasthan, SK - Sikkim, TN - Tamil Nadu, TR - Tripura, UP - Uttar Pradesh, WB - West Bengal. **A.** Bivariate LISA cluster map for any smoking prevalence versus any drinking prevalence. **B.** Bivariate LISA cluster map for cigarette smoking prevalence versus bidi smoking prevalence.

The results from bivariate LISA analysis showed that compared to univariate LISA, sizes of high-high and low-low clusters were smaller and the number of high-low and low-high outliers was higher (Figure 3A–B, Figure S3C–D). This indicates that the bivariate spatial correlations between smoking and drinking were not as strong as those found in the univariate analysis. The bivariate LISA results were supported by the bivariate Moran’s I results as most of the bivariate pairs had weak and non-significant results (Table 3). Nonetheless, it can be observed that for smoking versus drinking, high-high clusters exist in Himachal Pradesh, and low-low clusters exist in Gujarat and Maharashtra (Figure 3A). Cigarette smoking had the strongest spatial correlation with drinking (Figure S3C). For cigarette smoking versus bidi smoking, high-high clusters were found in West Bengal, and low-low clusters were found in Punjab, Maharashtra, and Orissa (Figure 3B). The low-high outliers found in the central states clearly showed that low prevalence of cigarette smoking coexisted with high prevalence of bidi smoking, and vice versa for the states of Kerala and Tamil Nadu. Overall, there was high prevalence for both cigarette and bidi smoking in West Bengal, and low prevalence for both smoking and drinking in Gujarat and Maharashtra (Table 4).

**Table 4:** Broad geographic patterns by regions across India for smoking and drinking variables.

Region	Variable (State name if applicable)	
	with high prevalence	with low prevalence
North	Any drinking (Himsachal Pradesh)	Any smoking (Punjab); Bidi smoking (Punjab)
Northeast	Any smoking; Cigarette smoking; Any drinking (Arunachal Pradesh, Sikkim)	n/a
East	Any smoking (West Bengal); Bidi smoking (West Bengal); Cigarette smoking (West Bengal)	Any smoking (Orissa); Bidi smoking (Orissa)
Central	Any smoking; Bidi smoking; Early smoking (before age 20)	Cigarette smoking
West	n/a	Any smoking (Maharashtra);Cigarette smoking (Gujarat, Maharashtra); Bidi smoking (Maharashtra); Early smoking (before age 20) (Maharashtra); Any drinking (Gujarat, Maharashtra)
South	Cigarette smoking (Kerala)	Any smoking (Tamil Nadu); Bidi smoking (Tamil Nadu)

We compared our results on smoking prevalences with the living respondents in the Million Death Study (MDS) [23] and adults interviewed in the Global Adult Tobacco Survey (GATS) 2009–2010 [3]. We found correlations of smoking prevalences between SFMS in 1998 and MDS in 2004 at the district level (any smoking: R^2^ = 0.58, p<0.01; cigarette smoking: R^2^ = 0.52, p<0.01; bidi smoking: R^2^ = 0.56, p<0.01), and high correlations between SFMS in 1998 and GATS in 2010 at the state level (any smoking: R^2^ = 0.67, p<0.01; cigarette smoking: R^2^ = 0.64, p<0.01; bidi smoking: R^2^ = 0.70, p<0.01).

## Discussion

Our results confirmed that smoking prevalence is significantly higher in the northeastern and central states, while it is significantly lower in Punjab, Maharashtra, and Orissa. These state level results of smoking are comparable to the findings from previous research studies [11], [12] and GATS [3]. Punjab had significantly lower smoking prevalence as the majority of its population practices Sikhism, which prohibits tobacco use [11], [12]. The high prevalence in the northeastern states is consistent with the GATS report [3]. Moreover, high prevalence has been reported among students in this region [35].

There was a high prevalence of any drinking in Himachal Pradesh, south Bihar, eastern Madhya Pradesh (presently Chhattisgarh state), and in the northeastern states (Sikkim and Arunachal Pradesh in particular). Many of these states have high proportion of tribal population [36] and liquor drinking is common among the tribal persons [37], as well as a low proportion of Muslim population [38], in whom the reported alcohol drinking prevalence is low [14], [39]. The low prevalence in Gujarat and Maharashtra could be attributed to the alcohol prohibition in these states, with Gujarat under complete prohibition (theoretically) on the production and sale of alcohol [7]. Surprisingly, we found that Tamil Nadu had low drinking prevalence even though other research has found high drinking prevalence [40], [41].

Our results showed the sub-state variations of smoking and drinking prevalence and the coexistence of high-high and low-low clusters with outliers within a state. For example, in Madhya Pradesh low-low clusters for cigarette smoking were found in the western and eastern part of the state, with high-low outliers found in the central part of the state. These spatial heterogeneities of tobacco and alcohol use at the sub-state levels were also found in previous studies [12], [14].

Cigarette smoking was high in the northeastern and the southern states, while bidi smoking was high in the central states and in West Bengal. These two smoking types are characterized by different users in India. Cigarette smoking is common among the educated and people with skilled occupations, while bidi smoking is common among the rural population, the urban poor, and the less educated [9], [42]. The relatively inexpensive bidis are more affordable and attractive to young people [43]. Our results show that the geographic patterns of bidi smoking closely resemble those of early smoking (before age 20). Control policies on bidi smoking thus may reduce smoking uptake among young people.

Since 1998, data has shown an increase in sales and the affordability of tobacco [44], despite the implementation of several tobacco control policies [45], [46]. Limited data suggested an increase in smoking prevalence, in particular among younger people [44]. Evidence also suggests that cigarettes are displacing bidis among younger males [47]. Meanwhile, increases in alcohol sales and use have been observed [5], [48], along with the more relaxed restrictions on consumption in many states [49]. For example, alcohol prohibition is slowly being liberalized and consumption is increasing in Maharashtra. The increase in popularity of both cigarette smoking and drinking required our attention as we found an association between the two. Previous studies also found complementarities between tobacco smoking and alcohol drinking among Indians [8]–[10]. Smoking cessation may enhance alcohol abstinence [50] and certain types of smoking cessation medication can help reduce the frequency of alcohol consumption [51]. Thus, local public health action on smoking might help reduce alcohol consumption, and vice versa. Tobacco and alcohol control is currently implemented at the state level [6], [16], and thus reliable monitoring of changes in consumption patterns and complementarities between cigarette smoking and alcohol drinking at the district level is required to enable effective control.

This study has several limitations. First, the SFMS was aimed to provide information representative at the state level [24], similar to the NFHS 1998–1999 [25] and GATS 2009–2010 [3]. But its large sample size yielded over 1 million individuals for this study, which provide sufficient number for stable district level analysis. Results from the kriging interpolations also confirmed the geographic patterns at the district level. Second, the SFMS was conducted over a decade ago and the current level of smoking and drinking among Indians may now differ. However, smoking prevalences in 1998 were correlated with prevalence in 2004 at the district level (SFMS *vs.* MDS) and with prevalences in 2010 at the state level (SFMS *vs.* GATS). Notwithstanding the different survey designs between SFMS and GATS (i.e. the use of household informants in SFMS and selected individuals in GATS), conclusions derived from our results at sub-state level are still likely to apply to the current Indian population. Third, the use of household informants may lead to under-reporting of smoking and drinking behaviours as they may not be aware of these consumption behaviours of the household residents (such as number of days alcohol was used), especially among younger males. Under-reporting could also be caused by the social desirability bias. For example, in states where prohibitions on tobacco and alcohol use were in effect, respondents may be less likely to report the smoking and drinking behaviours of their household residents. Fourth, our results are nationally representative and applicable to Indian males aged 30–69, which comprises the majority of smokers in India [11], [26]. The exclusion of women was reasonable as smoking and drinking prevalences among women remained low in the last decade [3], [11], [27], [28]. Similarly, the exclusion of six states (with 19% of study population) from the analysis of cigarette and bidi geographic patterns should not greatly alter the national results for the remaining 23 states. Finally, although the prevalence for the various smoking and drinking variables were not markedly different between the district level and postal code level study populations, it is possible that the 7% of records excluded in the postal code level were spatially clustered. Therefore, the kriging interpolations only provide the approximate distributions of smoking and drinking at the sub-district level.

## Conclusion

India has over 100 million current tobacco smokers and it accounts for approximately one-fifth of the world’s tobacco-related deaths [52], [53]. There is an urgent need of ongoing research to determine the current level of smoking in the population. Our study informs Indian tobacco control policy by filling the research gap of tobacco use and the use of cigarette and bidi smoking at the sub-state level in India. We found that cigarette smoking dominated the northeastern and southern states and bidi smoking dominated the central states, with early smoking (before age 20) closely resembling the geographic patterns of bidi smoking. We also identified high-burden areas of drinking and its spatial association with cigarette smoking. Finally, the sub-state variations of smoking and drinking require the attention of state policymakers. In order to control tobacco and alcohol use more effectively, state governments should implement the existing national acts of tobacco and alcohol control at the district level. Surveys that properly represent their consumption at the district level are recommended. Reductions in smoking may simultaneously reduce alcohol consumption, and vice versa.

## Supporting Information

Figure S1 **Cigarette smoking and bidi smoking prevalence at the district and postal code levels.** Prevalence at district level was age standardized using Census of India 2001 national population; prevalence at postal code level was estimated crude prevalence from kriging interpolation. State abbreviations: AP - Andhra Pradesh, AR - Arunachal Pradesh, AS - Assam, BR - Bihar, CH - Chandigarh, DD - Daman and Diu, DL - Delhi, DN - Dadra & Nagar Haveli, GA - Goa, GJ - Gujarat, HP - Himachal Pradesh, HR - Haryana, JK - Jammu & Kashmir, KA - Karnataka, KL - Kerala, MG - Meghalaya, MH - Maharashtra, MN - Manipur, MP - Madhya Pradesh, MZ - Mizoram, NL - Nagaland, OR - Orissa, PB - Punjab, PD - Pondicherry, RJ - Rajasthan, SK - Sikkim, TN - Tamil Nadu, TR - Tripura, UP - Uttar Pradesh, WB - West Bengal. **D.** Cigarette smoking prevalence at district and postal code levels. **E.** Bidi smoking prevalence at district and postal code levels.
(TIF)

Figure S2 **LISA cluster maps for cigarette smoking and bidi smoking prevalence at the district level.** State abbreviations: AP - Andhra Pradesh, AR - Arunachal Pradesh, AS - Assam, BR - Bihar, CH - Chandigarh, DD - Daman and Diu, DL - Delhi, DN - Dadra & Nagar Haveli, GA - Goa, GJ - Gujarat, HP - Himachal Pradesh, HR - Haryana, JK - Jammu & Kashmir, KA - Karnataka, KL - Kerala, MG - Meghalaya, MH - Maharashtra, MN - Manipur, MP - Madhya Pradesh, MZ - Mizoram, NL - Nagaland, OR - Orissa, PB - Punjab, PD - Pondicherry, RJ - Rajasthan, SK - Sikkim, TN - Tamil Nadu, TR - Tripura, UP - Uttar Pradesh, WB - West Bengal. **D.** LISA cluster map for cigarette smoking prevalence. **E.** LISA cluster map for bidi smoking prevalence.
(TIF)

Figure S3 **Bivariate LISA cluster maps.** State abbreviations: AP - Andhra Pradesh, AR - Arunachal Pradesh, AS - Assam, BR - Bihar, CH - Chandigarh, DD - Daman and Diu, DL - Delhi, DN - Dadra & Nagar Haveli, GA - Goa, GJ - Gujarat, HP - Himachal Pradesh, HR - Haryana, JK - Jammu & Kashmir, KA - Karnataka, KL - Kerala, MG - Meghalaya, MH - Maharashtra, MN - Manipur, MP - Madhya Pradesh, MZ - Mizoram, NL - Nagaland, OR - Orissa, PB - Punjab, PD - Pondicherry, RJ - Rajasthan, SK - Sikkim, TN - Tamil Nadu, TR - Tripura, UP - Uttar Pradesh, WB - West Bengal. **C.** Bivariate LISA cluster map for cigarette smoking prevalence versus any drinking prevalence. **D.** Bivariate LISA cluster map for bidi smoking prevalence versus any drinking prevalence.
(TIF)

Appendix S1 **Explanation on age standardization, kriging method, Moran’s I and LISA statistics.**
(DOC)
