# Supplementary figures and images for: Geospatial Analysis on the Distributions of Tobacco Smoking and Alcohol Drinking in India
Source: PLoS One. 2014 Jul 15;9(7):e102416. doi: 10.1371/journal.pone.0102416 (PMC4099149; doi:10.1371/journal.pone.0102416)

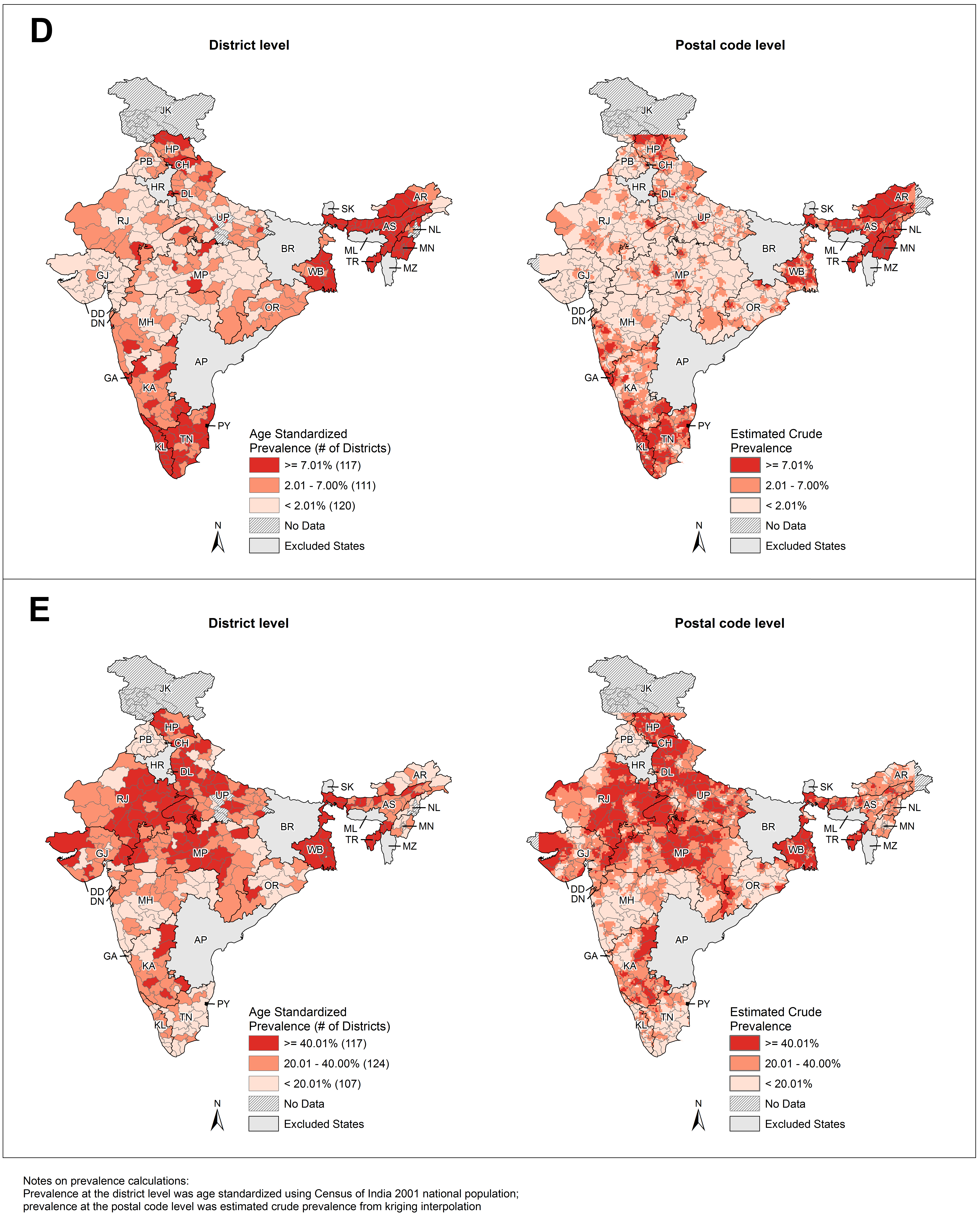

Supplement: Figure S1 — Cigarette smoking and bidi smoking prevalence at the district and postal code levels. Prevalence at district level was age standardized using Census of India 2001 national population; prevalence at postal code level was estimated crude prevalence from kriging interpolation. State abbreviations: AP - Andhra Pradesh, AR - Arunachal Pradesh, AS - Assam, BR - Bihar, CH - Chandigarh, DD - Daman and Diu, DL - Delhi, DN - Dadra & Nagar Haveli, GA - Goa, GJ - Gujarat, HP - Himachal Pradesh, HR - Haryana, JK - Jammu & Kashmir, KA - Karnataka, KL - Kerala, MG - Meghalaya, MH - Maharashtra, MN - Manipur, MP - Madhya Pradesh, MZ - Mizoram, NL - Nagaland, OR - Orissa, PB - Punjab, PD - Pondicherry, RJ - Rajasthan, SK - Sikkim, TN - Tamil Nadu, TR - Tripura, UP - Uttar Pradesh, WB - West Bengal. D. Cigarette smoking prevalence at district and postal code levels. E. Bidi smoking prevalence at district and postal code levels. (TIF) [file pone.0102416.s001.tif]

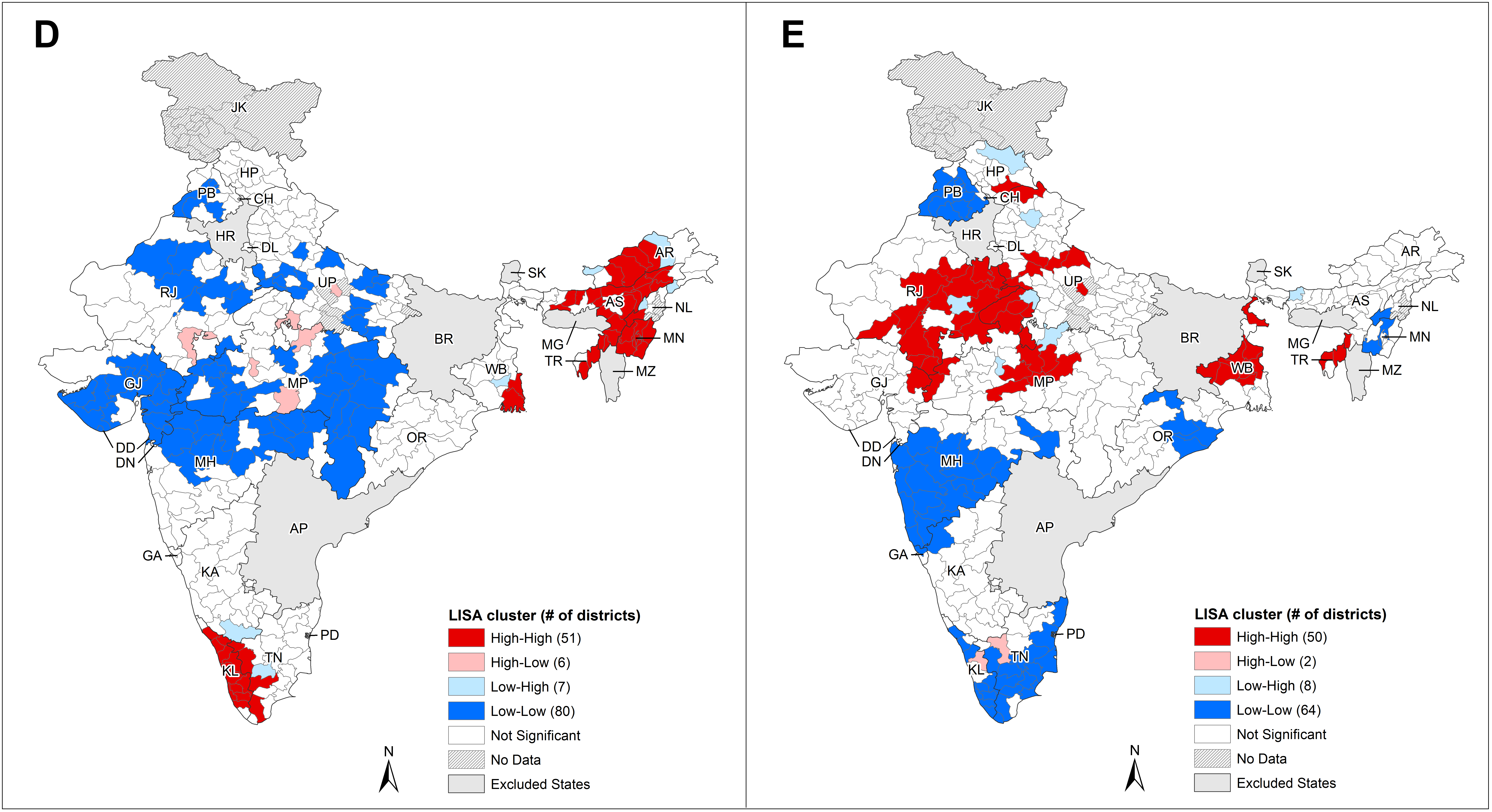

Supplement: Figure S2 — LISA cluster maps for cigarette smoking and bidi smoking prevalence at the district level. State abbreviations: AP - Andhra Pradesh, AR - Arunachal Pradesh, AS - Assam, BR - Bihar, CH - Chandigarh, DD - Daman and Diu, DL - Delhi, DN - Dadra & Nagar Haveli, GA - Goa, GJ - Gujarat, HP - Himachal Pradesh, HR - Haryana, JK - Jammu & Kashmir, KA - Karnataka, KL - Kerala, MG - Meghalaya, MH - Maharashtra, MN - Manipur, MP - Madhya Pradesh, MZ - Mizoram, NL - Nagaland, OR - Orissa, PB - Punjab, PD - Pondicherry, RJ - Rajasthan, SK - Sikkim, TN - Tamil Nadu, TR - Tripura, UP - Uttar Pradesh, WB - West Bengal. D. LISA cluster map for cigarette smoking prevalence. E. LISA cluster map for bidi smoking prevalence. (TIF) [file pone.0102416.s002.tif]

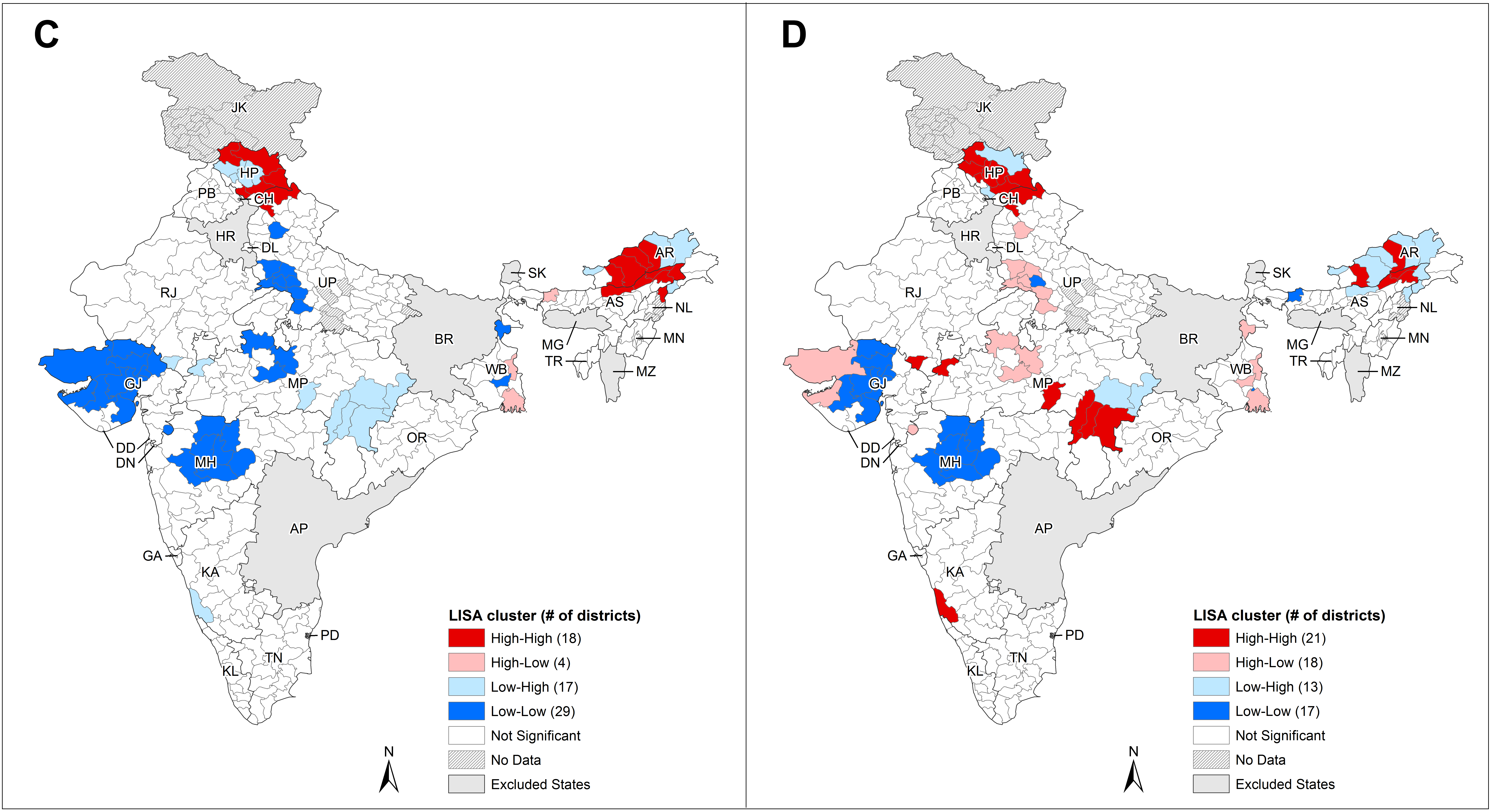

Supplement: Figure S3 — Bivariate LISA cluster maps. State abbreviations: AP - Andhra Pradesh, AR - Arunachal Pradesh, AS - Assam, BR - Bihar, CH - Chandigarh, DD - Daman and Diu, DL - Delhi, DN - Dadra & Nagar Haveli, GA - Goa, GJ - Gujarat, HP - Himachal Pradesh, HR - Haryana, JK - Jammu & Kashmir, KA - Karnataka, KL - Kerala, MG - Meghalaya, MH - Maharashtra, MN - Manipur, MP - Madhya Pradesh, MZ - Mizoram, NL - Nagaland, OR - Orissa, PB - Punjab, PD - Pondicherry, RJ - Rajasthan, SK - Sikkim, TN - Tamil Nadu, TR - Tripura, UP - Uttar Pradesh, WB - West Bengal. C. Bivariate LISA cluster map for cigarette smoking prevalence versus any drinking prevalence. D. Bivariate LISA cluster map for bidi smoking prevalence versus any drinking prevalence. (TIF) [file pone.0102416.s003.tif]
