# Supplementary material for: Geospatial Analysis on the Distributions of Tobacco Smoking and Alcohol Drinking in India
Source: PLoS One. 2014 Jul 15;9(7):e102416. doi: 10.1371/journal.pone.0102416 (PMC4099149; doi:10.1371/journal.pone.0102416)
Supplement: Appendix S1 — Explanation on age standardization, kriging method, Moran’s I and LISA statistics. (DOC) [file pone.0102416.s004.doc]

**Appendix S1**

**Explanation on age standardization, kriging method, Moran’s I and LISA statistics**

**Kriging**

Kriging is a technique that generates an estimated interpolation surface from a set of data points . This technique incorporates inference from the spatial structure of data points to derive estimations at unmeasured locations. The kriging interpolation method involves three steps to generate an interpolation surface: 1) to determine the variogram which measures the spatial variation and autocorrelation for the variable of interest, 2) to fit the variogram with a theoretical model (such as spherical, exponential models) in order to provide the necessary input parameters for kriging interpolation; and 3) to make an interpolation using the fitted model, which can be depicted by this general formula:

$$\hat{Z}(x_{0})=\sum_{i=1}^{n} \lambda_{i}Z(x_{i})$$

where $\hat{Z}(x_{0})$is the interpolated value at location *x_0_*, *Z*(*x_i_*) is the measured valued at location *x_i_*, n is the number of neighbours within the search neighbourhood used for the estimation, and *λ_i_* is weighting function based on the parameters of the variogram model (see for more detail).

Each household member of the SFMS study population was associated with an SRS unit code. However, because spatial locations of the SRS units were not available, the study population at the SRS unit level had to be aggregated to the Indian postal code locations (a total of 4154 postal codes). Only crude prevalences were calculated for the smoking and drinking variables at these Indian postal code locations, as age standardizations could not provide reliable results due to the small number of observations at some postal code locations. The kriging procedure used the calculated crude prevalence at the postal code points to create an interpolation surface for the entire country.

**Moran’s I and LISA**

Moran’s I is an index that measures the “global” spatial autocorrelation of variable values over the entire dataset . It is given by

where *n* is the total number of spatial features (i.e. districts), *x_i_* is the value for the spatial feature *i*, $\bar{x}$ is the mean value of all spatial features, *j* are neighbouring features, *w_ij_* is the spatial weight between feature *i* and *j* in row standardized form. In this study, we defined neighbours as the districts that share either a common border or a vertex with a given district (*x_i_*). An index score derived from the calculation indicates the strength of spatial autocorrelation of the variable values, with a score of zero indicating no spatial autocorrelation, a score of 0.3 or more indicating a relatively strong positive autocorrelation (i.e. clustering of features of similar values), and -0.3 or less indicating a relatively strong negative autocorrelation (i.e. neighbouring features tend to have dissimilar attribute values) .

The LISA statistic gives an indication of the extent of significant spatial clustering of similar or dissimilar values around a spatial feature . It is given by

The parameters for the LISA statistics are same as those in Moran’s I. In fact, the sum of the LISA statistics for all spatial features is proportional to the global Moran's I. A positive *I_i_* value indicates spatial clustering of similar values around a spatial feature, and negative values indicate a clustering of dissimilar values around a spatial feature. Four types of spatial associations can be derived from this statistic, with high-high (HH) and low-low (LL) types for spatial clustering of similar values, and high-low (HL) and low-high (LH) types for spatial clustering of dissimilar values, that is, spatial outliers .

Two types of Moran’s I and LISA analyses were performed: univariate and bivariate analyses. Univariate analysis measures the correlation of neighbouring values around a district for the same variable, whereas bivariate analysis measures the correlation between the value of one variable for the observation and neighbouring values of a second variable. Using the district-level age standardized prevalence (see below) as data values, we performed univariate Moran’s I and LISA to examine the spatial autocorrelation and spatial clusters, respectively, for each smoking and drinking variable. We used the bivariate Moran’s I to examine the spatial autocorrelation between smoking and drinking, as well as between the smoking types; we used the bivariate LISA to identify the spatial clusters. Due to the nature of the bivariate Moran’s I and LISA statistics, slightly different but valid results are produced depending on which variable is used as the dependent variable (i.e. value at the spatial feature) and the spatial lag variable (i.e. values from neighbouring features) . In this study, smoking was chosen as the dependent variable to compare against drinking prevalence from neighbouring districts, while cigarette smoking was used as the dependent variable to compare against bidi smoking prevalence from neighbouring districts. Preliminary analyses were also conducted using drinking and bidi smoking as the dependent variables. These results gave similar conclusions (data not shown).

A permutation approach was used to assess the statistical significance of the results . The procedure involves recalculating the statistics many times by randomly permuting the data values in the dataset to generate a spatially random reference distribution (for LISA analysis, value at location *i* is held fixed in the random permutation ). The observed statistic based on the original dataset is compared against this reference distribution to assess the statistical significance. We used 49999 permutations to derive the reference distribution. A significance level of 0.05 was used for the final results.

**Age standardized prevalence**

Age standardized prevalence (ASP) allowed the comparison of smoking and drinking prevalences across districts. Age standardized smoking and drinking prevalences for each district were then calculated using the national population from the Census of India 2001 , based on five-year age categories (i.e., a total of eight age categories between the ages 30 and 69 years). ASP is defined as:

$$ASP=\sum_{i}^{n} (r_{i}\times w_{i});w_{i}=\frac{p_{i}}{p_{total}}$$

where *n* is the total number of age categories, *r_i_* is the smoking/drinking crude prevalence and *w_i_* is the weight for the age category *i*, which is derived from the division of census population in age category *i* (*p_i_*) by the census national population (*p_total_*).

**References**

1. O'Sullivan D, Unwin D (2010) Geographic Information Analysis. Hoboken, New Jersey: John Wiley & Sons, Inc.

2. Oliver MA, Webster M (1990) Kriging: a method of interpolation for GIS. International Journal of Geographical Information Systems 4: 313-332.

3. Pfeiffer DU, Robinson TP, Stevenson M, Stevens KB, Rogers DJ, et al. (2008) Spatial Analysis in Epidemiology: Oxford University Press.

4. Anselin L (1995) Local Indicators of Spatial Association - Lisa. Geographical Analysis 27: 93-115.

5. Anselin L (2005) Exploring Spatial Data with GeoDa: A Workbook. Spatial Analysis Laboratory, Department of Geography University of Illinois and Center for Spatially Integrated Social Science, IL, USA.

6. Census of India (2001) Census Reference Tables, C – Series: Social and Cultural Tables. New Delhi: Registrar General of India, Government of India.
